# Testing whether barriers to a hypothetical screening test affect unrelated perceived benefits and vice versa: A randomised, experimental study

**DOI:** 10.1016/j.pec.2016.09.007

**Published:** 2017-02

**Authors:** Alex Ghanouni, Ella Nuttall, Jane Wardle, Christian von Wagner

**Affiliations:** Department of Epidemiology and Public Health, University College London, London, UK

**Keywords:** Public health, Preventive medicine, Screening, Decision making, Risk communication, Risk perception, Affect, Emotion, Cognitive biases, Survey methods

## Abstract

**Objective:**

Determine whether (fictitious) health screening test benefits affect perceptions of (unrelated) barriers, and barriers affect perceptions of benefits.

**Methods:**

UK adults were recruited via an online survey panel and randomised to receive a vignette describing a hypothetical screening test with either high or low benefits (higher vs. lower mortality reduction) and high or low barriers (severe vs. mild side-effects; a 2 × 2 factorial design). ANOVAs compared mean perceived benefits and barriers scores. Screening ‘intentions’ were compared using Pearson’s χ^2^ test.

**Results:**

Benefits were rated less favourably when barriers were high (mean: 27.4, standard deviation: 5.3) than when they were low (M: 28.5, SD: 4.8; p = 0.010, partial η^2^ = 0.031). Barriers were rated more negatively when benefits were low (M: 17.1, SD: 7.6) than when they were high (M: 15.7, SD: 7.3; p = 0.023, partial η^2^ = 0.024). Most intended to have the test in all conditions (73–81%); except for the low benefit-high barrier condition (37%; p < 0.0005; N = 218).

**Conclusions:**

Perceptions of test attributes may be influenced by unrelated characteristics.

**Practice implications:**

Reducing screening test barriers alone may have suboptimal effects on perceptions of barriers if benefits remain low; increasing screening benefits may not improve perceptions of benefits if barriers remain high.

## Highlights

• Perceptions of test barriers were more negative when unrelated screening benefits were low.
• Perceptions of screening benefits were less positive when unrelated test barriers were high.
• Screening intentions were markedly lower for a test with both high barriers and low benefits.

## 1 Introduction

Screening is an important public health strategy for reducing cancer mortality and incidence. There is potential to improve population health by increasing uptake of available screening tests but people’s willingness to undergo them typically requires them to accept some short-term burden and some level of risk in exchange for a degree of potential health benefit in the relatively distant future. Much informative research has been carried out on how invitees perceive benefits and barriers of screening in order to address the policy goal of improving uptake (and satisfaction with screening services in general).

Studies in this area have often been guided by psychological theories which assume implicitly that perceptions of barriers and benefits are independent. For example, the Health Belief Model [1] includes benefits and barriers as discrete ‘constructs’ that are often analysed separately (e.g. [2], [3]). Similar conceptual and analytical approaches are also apparent in less theoretically-oriented research (e.g. where perceived barriers are examined without assessment of perceived benefts [4]).

However, this assumption may not be not true; appraisals of barriers may be less negative when benefits are high vs. when they are low (and likewise for perceptions of benefits when barriers are low vs. when they are high), even when those benefits are objectively unrelated. Previous research provides several theoretical bases for this hypothesis. Most notably, much research has found evidence that perceptions can be systematically ‘irrational’ in the context of evaluating whether to carry out a given health-related behaviour. For example, one cognitive shortcut known as the ‘affect heuristic’ suggests that individuals do not necessarily carry out separate appraisals of the favourable and unfavourable characteristics of a behaviour and evaluate the balance. Instead, both aspects are evaluated together, in the context of a shared ‘pool’ of feeling or emotion (i.e. ‘affect’). That is, where an affective response towards a behaviour is positive, desirable characteristics (e.g. health benefits) are judged to be high and aversive characteristics (e.g. risks or barriers) are judged to be low, whereas the opposite applies if the affective response is negative [5], [6]. Affect may also lead to interrelatedness in other ways, such as through directing attention to particular information: positive feelings towards screening may increase the extent to which benefits are focused on and decrease the extent to which barriers are considered [7]. There are various other rationales for this hypothesised interaction, some of which are more cognitive in nature, such as halo effects (in which characteristics of a behaviour are evaluated in terms of general attitudes towards it) and efforts to maintain cognitive consistency (i.e. people may attempt to avoid ‘incompatible’ views where favourable aspects of a behaviour are seen as positive while unfavourable aspects would be seen as negative simultaneously) [8].

Irrespective of the psychological underpinnings, empirical evidence provides some support for this hypothesis; cross-sectional studies have often found that perceptions of screening test benefits and barriers are negatively correlated [9], [10], [11], [12]. However, to our knowledge, no experimental studies have tested this hypothesis of interrelatedness directly, meaning that their applicability to screening policy is limited. It is important to investigate this relationship because efforts to improve screening uptake based on addressing invitees’ stated barriers will have limited success if they are proxies for negative perceptions regarding other aspects of screening.

This study used an experimental design to test whether modifying test barriers affected perceptions of conceptually unrelated benefits, and vice versa. Participants were allocated at random to receive information regarding a screening test with high or low benefits, and high or low barriers, in the context of a hypothetical disease with similarities to cancer. Perceived benefits and barriers were then compared between conditions in order to test i) whether perceptions of benefits were lower when barriers were higher, and ii) whether perceptions of barriers were higher when benefits were lower. Intention to have the hypothetical test was also compared between conditions as an exploratory analysis of how the manipulation might affect actual screening behaviour.

## 2 Materials and methods

### 2.1 Participants

Recruitment was through Survey Sampling International (SSI, London, UK), a company which curates a panel of members of the UK general population who are offered small rewards (such as air miles) to participate in online surveys. Respondents to the initial email invitation from SSI were asked their age at the start of the survey and excluded if they were younger than 25 or older than 75 years (i.e. ineligible for cancer screening in the UK). A software algorithm applied stratified sampling to ensure that the sample resembled the general adult UK population in terms of age; one third of the sample were aged 25–39 years, one third were aged 40–54 years, and one third were aged 55–75 years.

### 2.2 Design and measures

#### 2.2.1 Manipulations

This study consisted of a 2 × 2 between-subjects experimental design. Participants were invited to complete one of four versions of a survey, randomly determined by a software algorithm. After confirming eligibility, they were shown a vignette consisting of information on the high incidence (33%) of a hypothetical illness that was amenable to screening (‘Rogan’s disease’), the rationale for screening, and the extremely high mortality risk in the absence of a screening test (only 100 in 1000 would survive). Participants were also given a description of a set of practicalities for a hypothetical hospital-based screening test, designed to resemble Computed Tomography (a screening test based on x-rays). This test can include an intravenous dye that carries a small risk of an adverse reaction, consisting of nausea and vomiting [13], the severity of which was manipulated as a screening test barrier (*“severe nausea and regular vomiting for 3 days”*; *“mild nausea and occasional vomiting for 5 *minutes*”*). The specific types of benefits and barriers were selected with the aim of being realistic, understandable, and plausible to participants, potentially influential on their intentions to have the test, as well as being fundamentally unrelated (as opposed to e.g. false positive and false negative results).

**Fig. 1:**
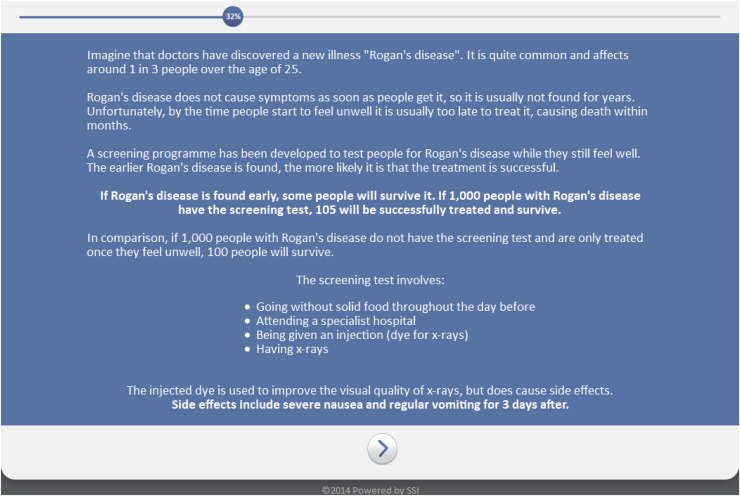
An example of a complete information vignette (low benefit; high barrier).

Information on the degree of benefit was provided in terms of the mortality risk after undergoing a screening test. This was manipulated to equal either a large or a comparatively small reduction in mortality risk (900 per 1000 with Rogan’s disease who underwent screening would survive; 105 per 1000 would survive). Fig. 1 contains an example of a complete vignette. Levels of barriers and benefits were designated “high” and “low” for convenience.

#### 2.2.2 Comprehension checks

Participants were asked three multiple choice questions with four response options to assess whether they correctly recalled the relevant information on mortality risk in the presence or absence of screening (e.g. *“If 1000 people with Rogan’s disease are not screened and only treated once they feel unwell, how many people will be successfully treated and survive?”*: 100 people; 105 people; 500 people; 900 people), and information on the severity of the adverse reaction. Responses were coded as either correct or incorrect based on the allocated condition.

#### 2.2.3 Perceived benefits and barriers scales

Primary outcomes were assessed by seven items measuring perceived benefits (e.g. *“Having the screening test would increase my chances of surviving Rogan’s disease”*) and seven items assessing perceived barriers of the screening test (e.g. *“the side-effects would be too uncomfortable”*). Response options consisted of a five-point Likert scale ranging from *“strongly disagree”* to *“strongly agree”*. Items were adapted from existing measures [14], [15], [16] and demonstrated high internal consistency (Cronbach’s α: 0.89 and 0.96, respectively). Responses were scored from one to five with higher scores representing more positive perceptions of benefits and more negative perceptions of barriers, as applicable. Scores for individual items were summed to create two overall scale scores for each participant (each out of 35).

#### 2.2.4 Perceived risk

Participants were asked about perceived risk using an adaptation of a previously designed measure [17] with six response options (*“If I didn*’t *have the screening test, I think my chances of dying from Rogan’s disease would be…”: Almost zero; very small; moderate; large; very large; almost certain*).

#### 2.2.5 Self-efficacy

A five-item assessment of self-efficacy (e.g. *“How confident are you that...You could find the time to have the screening test?”*), with four response options ranging from “very confident” to “not at all confident”, was adapted from a previous measure [18] and this also had high internal consistency (Cronbach’s α = 0.93). Responses were scored from one to four; higher scores represented greater self-efficacy and were summed to create an overall scale score for each participant (out of 20).

#### 2.2.6 Screening intention

Intention to participate in screening was assessed using an ad-hoc item: *“Imagine the NHS just sent you a letter, inviting you to be screened for Rogan’s disease. Would you attend the screening test?”*). Response options consisted of *“yes”*, *“no”*, and *“don’t know”*.

#### 2.2.7 Demographics

The survey ended with items assessing demographic characteristics, including gender, first language, and markers of socioeconomic status. A previously used method was used to derive an overall measure of socioeconomic status, based on responses to questions on home and vehicle ownership, and education [19]: One point was counted for living in rented accommodation, no vehicle ownership, and no formal qualifications; higher scores indicated greater deprivation. Previous participation in the three cancer screening programmes that exist in the UK were also assessed (cervical, breast and colorectal; questions were tailored by age and gender so that ineligible participants did not see irrelevant questions).

At the end of the survey, participants were able to request a summary copy of the study results. An example of the full survey is included in Appendix A.^1^

### 2.3 Piloting

Prior to data collection, the manipulations for high and low benefits and barriers were tested in two waves, consisting of 32 and 26 participants, respectively. Each wave aimed to ensure that participants in the main study would discriminate between high and low levels of the two independent variables. In particular, it was assumed that participants would perceive high benefits from even very few lives saved through screening, which would have led to ceiling effects that reduced the perceived differences between high and low levels. Perceived benefits and barriers of several possible manipulations were assessed using two ad-hoc items and results were used to select levels that were likely to generate the largest possible differences while still being believable to participants. As an example, the first wave of piloting compared perceived benefits of 800 vs. 200 people surviving following screening (relative to 100 people surviving without screening). Notwithstanding the small sample size, scores differed in the predicted direction but only by a small amount. Consequently, the second wave of piloting amended the number of lives saved to 900 vs. 105, which was associated with a larger apparent difference in perceived benefits scores. The first wave also compared perceived barriers of an alternative to test side-effects (travel time to the hospital: 20 min vs. 2 h). Similar to perceived benefits, scores differed in the predicted direction but to a smaller degree than the side-effect manipulation.

The second wave of piloting also assessed performance of the items adapted from previously used measures of perceived benefits and barriers, in order to gauge reliability prior to administering the survey to a larger sample.

### 2.4 Analysis

**Fig. 2:**
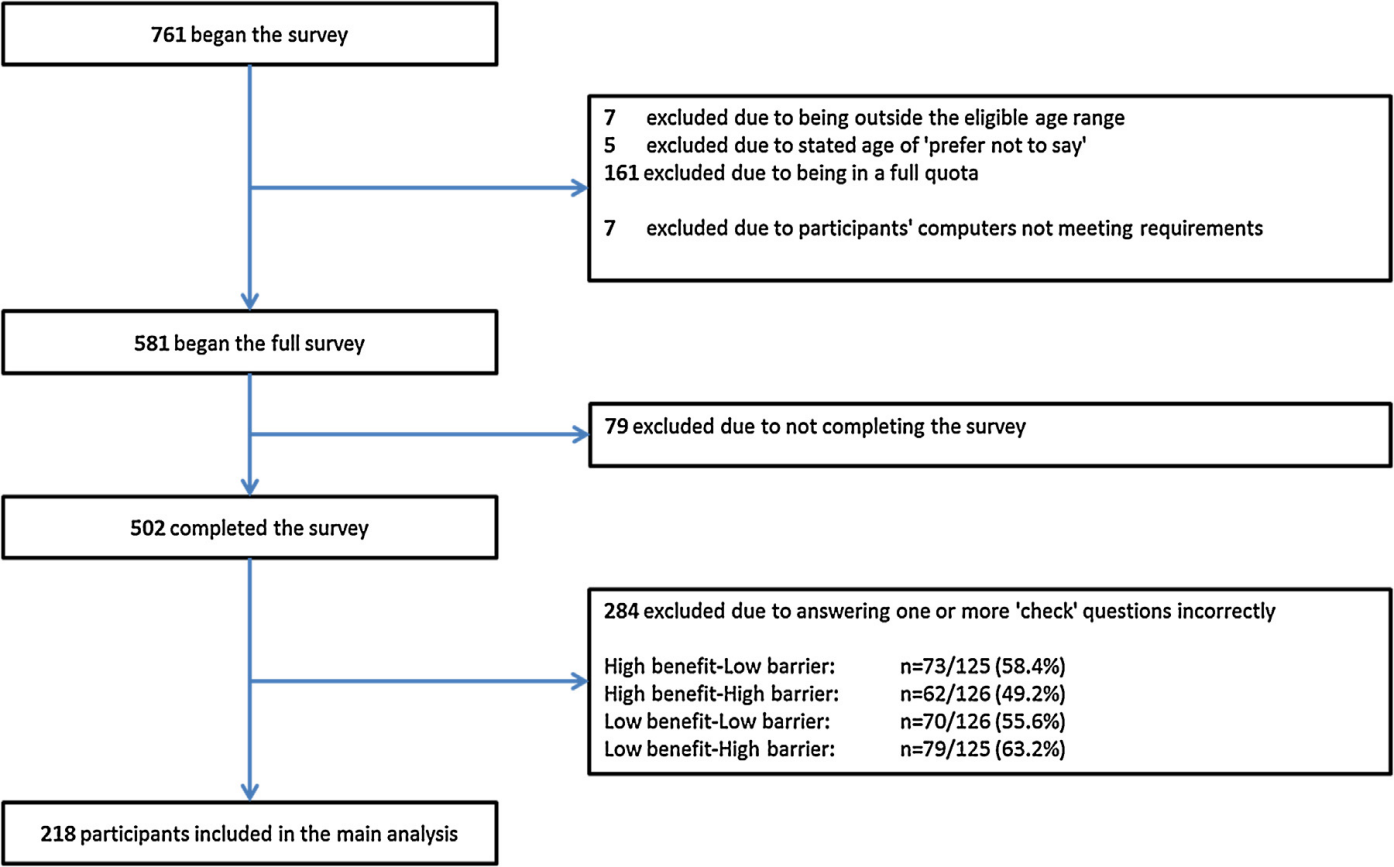
Flow of participants through the study.

Data were analysed using SPSS version 21 for Windows (IBM, Armonk, NY, USA). Participants answering one or more comprehension questions incorrectly were assumed to be insufficiently engaged with the survey and excluded from the analysis (Fig. 2). Descriptive statistics were used to illustrate frequencies and proportions for sample characteristics.

Parametric assumptions of data relating to perceived benefits and barriers (normally distributed residuals and homogeneity of variance) were tested and met. Hence, the primary analysis comprised two-way ANOVAs, one in which the dependent variable consisted of overall perceived benefit score and one in which the dependent variable was overall barrier score. In each ANOVA, independent variables consisted of benefit condition (high or low), barrier condition (high or low) and a benefit × barrier interaction term. Age-band (25–35, 40–54, and 55–75 years) was included to account for any effects of stratified sampling. A sensitivity analysis was carried out in which the age band variable was omitted; results did not differ meaningfully and so are not reported here. An exploratory analysis of screening intentions (proportions intending to be screened vs. not intending vs. did not know) compared responses across the four conditions using a Pearson’s χ^2^ test. Standardised residuals (i.e. z-scores based on the difference between observed and expected frequencies) were used to test for differences in proportions between any given pair of conditions.

### 2.5 Required sample size and hypotheses

The survey was ‘soft-launched’ and recruitment paused after 138 participants had completed the study in order to generate a preliminary estimate of mean square error for the dependent variables (necessary to calculate effect size). Since there is a direct conceptual link between perceptions of benefits and the actual magnitude of benefits, but not the actual magnitude of barriers, it was assumed that there would be a larger effect of manipulating benefits on perceived benefits than that of manipulating barriers. Likewise, manipulating barriers was expected to have a larger effect on perceived barriers than manipulating benefits. Calculations were based on a five-point difference for the effects of conceptually linked manipulations and a three-point difference for conceptually unrelated manipulations. Based on the initially observed mean square errors, it was estimated that a total of 204 participants would be required (51 participants per condition; 80% power, α = 0.05).

## 3 Results

### 3.1 Sample characteristics

The flow of participants through the study is presented in Fig. 2. After exclusions, 218 participants were included in the main analysis. Across the whole sample, participants had a mean age of 48.6 years (standard deviation: 13.6), 52.8% were female (n = 115), 86.7% (n = 189) were white British, and 96.3% (n = 210) spoke English as a first language. The majority of screening-eligible participants reported previous experience of testing, ranging from 73.6% for CRC screening to 87.9% for breast screening. All demographic and other background characteristics are presented in Table 1.

### 3.2 Effects of manipulating barriers (and manipulating benefits) on perceived benefits

As expected, manipulating benefits had an effect on perceived benefits, and in the predicted direction (F(1,212) = 55.25, p < 0.0005), providing an indication that the manipulation was successful (Mean: 30.0, standard deviation: 4.0 vs. M: 25.6, SD: 5.1 for high vs. low benefits, respectively). The primary hypothesis that increasing barriers also reduced perceived benefits was also supported (F(1,212) = 6.81, p = 0.010; M: 28.5, SD: 4.8 vs. M: 27.5, SD: 5.3 for low vs. high barriers, respectively). As predicted, the effect of manipulating barriers was smaller (partial η^2^ = 0.031) than that of manipulating benefits (partial η^2^ = 0.207). In terms of the effects of the interaction term, there was only weak evidence against the null hypothesis (p = 0.137).

### 3.3 Effects of manipulating benefits (and manipulating barriers) on perceived barriers

The manipulation of barriers was also successful; perceived barrier scores were higher when barriers were high (F(1,212) = 51.03, p < 0.0005; M: 19.6, SD: 7.6 vs. M:13.1, SD: 5.7). Consistent with the main hypothesis, increasing benefits also reduced perceived barriers (F(1,212) = 5.23, p = 0.023; M: 17.1, SD: 7.6 vs. M: 15.7, SD: 7.3 for low vs. high benefits, respectively). Again, this effect was smaller (partial η^2^ = 0.024) than that of manipulated barriers (partial η^2^ = 0.194). There was weak evidence against the null hypothesis with respect to the effects of the interaction term (p = 0.159). Table 2 reports means and standard deviations for perceived benefit and barrier scores for each of the four conditions.

### 3.4 Screening intentions

**Fig. 3:**
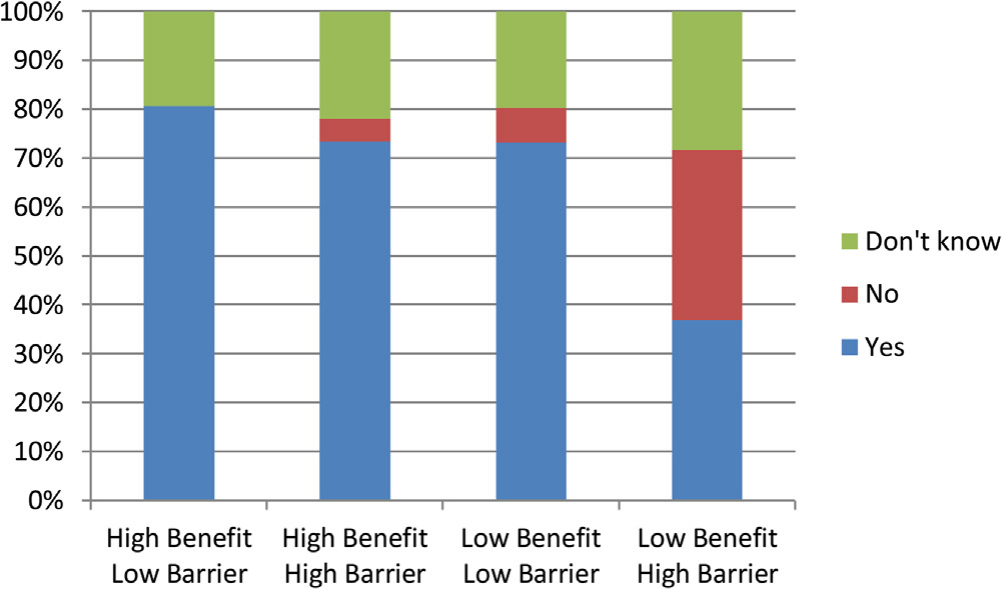
Proportions of screening intention responses across each condition.

There was strong evidence against the null hypothesis of equal proportions of intention categories across conditions (χ^2^(6) = 43.26, p < 0.0005). Follow-up analyses of standardised residuals provided evidence that a greater proportion of participants did not intend to be screened in the low benefit-high barrier condition, (34.8% vs. 0.0%–7.1%; z = − 2.5; p < 0.05), and a smaller proportion did intend to be screened (37.0% vs. 73.2%–80.8% z = 5.1; p < 0.01). In addition, fewer participants did not intend to be screened in the high benefit-low barrier condition (0.0% vs. 4.7%–34.8%; z = − 2.3; p < 0.05). Proportions of participants responding with *“don*’*t know”* were comparable between all conditions (19.2%–21.9%; Fig. 3).

## 4 Discussion and conclusion

### 4.1 Discussion

These findings provide evidence that screening attributes are not appraised independently but jointly, and manipulating one affects evaluations of the other. Our results build on cross-sectional studies that have demonstrated a negative correlation between benefits and barriers of cancer screening tests [9], [10], [11], [12] by showing that to some extent these correlations are likely to be due to a degree of interrelatedness between the two characteristics. This study also found that the large majority of participants stated that they would have the test in three of the four conditions. However, there was a marked difference in the worst condition (low benefits, high barriers), with a greater proportion stating that they would not have the test. This exploratory analysis offers some indication that barriers and benefits might interact in a way that influences screening uptake. Further research would also be necessary to understand how intentions (and ultimately actual uptake) relate to the observed interaction.

Although there is evidence that barriers and benefits are good predictors of behaviour when assessed individually [1], [20], there has been extensive criticism of the assumption made by the Health Belief Model that they have simple additive effects [1], [21]. In this respect, the present findings support researchers’ recommendations for alternative approaches that examine moderation among variables [1]. Our results further suggest that a degree of caution is warranted regarding research that aims to identify specific barriers to cancer screening without simultaneously addressing perceptions of benefits [4]: The issues that participants raise as important barriers to screening may be proxies for being unconvinced about the benefits [22], [23]. One further implication of these results is that screening tests with greater barriers might also elicit less positive perceptions of benefits. For example, flexible sigmoidoscopy screening for colorectal cancer (CRC) involves an invasive, internal examination and an inconvenient bowel preparation, which might diminish the effectiveness of interventions to improve uptake that aim solely to communicate its efficacy in terms of reducing CRC incidence and mortality. Conversely, these findings suggest that there may be potential to improve perceptions of screening test benefits by reducing barriers (and vice versa). As a practical example, as the Bowel Cancer Screening Programme in England replaces one method of stool testing with a less inconvenient alternative [24], this reduced inconvenience may lead to more favourable appraisals of the test’s capability to reduce mortality.

This study has limitations. The context of screening for a hypothetical illness allowed benefits and barriers to be manipulated freely, to the point that participants could discriminate between the two levels of each independent variable. However, the implications for practice with respect to real screening contexts are undetermined. It is notable that pilot work found similar benefit scores for even large differences in mortality reduction. The small observed effects may not apply to real screening contexts in which differences between tests are subtler. In addition, participants were excluded if they answered one of the three ‘comprehension check’ questions incorrectly, despite assistance offered to help them respond correctly. This approach aimed to exclude participants from the analysis if they had not read the relevant information and so were not sufficiently engaged with the study. However, it might have also resulted in a sample that was more numerate or literate than the general population. The proportion of exclusions was also greater in the low benefit-high barrier condition.

This study tested whether barriers affected perceptions of benefits and vice versa. However, it did not aim to test whether any particular psychological mechanism underpinned this relationship. The findings are consistent with the presence of an affect heuristic [5], which has been used to explain similar effects in appraisals of other technologies [6], but it is also consistent with various alternative explanations such as directing attention towards particular kinds of information [7], a halo effect, and attempts to avoid cognitive dissonance [8]. Further studies would be necessary to explore these possibilities. For example, subsequent studies could use a similar design but include measures of emotion in order to test for affective explanations. In the first instance, it would be important to test whether the effects of absolute barriers and benefits on unrelated outcomes were mediated via *perceived* barriers and benefits, respectively. Further research that uses these approaches would make a greater contribution to psychological theory.

Other areas for further research relate to the specific manipulations used: The set of benefits and barriers manipulated in the present study were selected following pilot work that aimed to maximise the chances of observing the hypothesised effect while still being believable to participants. This effect may not necessarily have been apparent with other benefits or barriers (e.g. one of our original tested barriers of travel distance to the hospital, which appeared to elicit smaller differences in perceived barriers between longer and shorter journey times than in the case of the side-effects attribute). However, characteristics of real screening tests are complex and multifactorial. Benefits can be medical and psychological; barriers can also be psychological as well as practical [21]. It may be particularly valuable to policy makers to determine the effects of manipulating specific characteristics of screening tests. For example, the risk of overdiagnosis in the case of breast cancer screening is the subject of intense debate [25] since it results in unnecessary treatment and the psychological harms of a cancer diagnosis. Overdiagnosis may be perceived more negatively by screening invitees than the practical barriers described in this study. Furthermore, it is often unfeasible to change real characteristics of screening tests but it is much easier to alter information in screening invitations. For example, different degrees of emphasis can be placed on information about barriers or benefits (e.g. by giving them greater prominence within an invitation leaflet, or by reiterating them in a leaflet summary). Manipulating these characteristics may increase or decrease some of the effects observed here. Moreover, the results of this study suggest that manipulating both attributes would have more than just an additive effect.

### 4.2 Conclusion

We found evidence that manipulating barriers of a screening test influenced perceived benefits and that manipulating benefits influenced perceived barriers. Future research should test the possible underlying psychological mechanisms and investigate the extent to which these findings generalise to real screening contexts. This would inform policy makers in their efforts to improve the balance of screening barriers and benefits in order to increase uptake.

## Conflicts of interest

None declared.

## Ethical approval

Ethical approval was granted by the UCL Research Ethics Committee (5791/001).

## Funding

The current study was supported by a programme grant from Cancer Research UK awarded to Prof Jane Wardle (C1418/A14134). Cancer Research UK was not involved in the design of this study; the collection, analysis, or interpretation of the results; in the writing of the manuscript; or in the decision to submit for publication.

## Informed consent and patient details

I confirm all patient/personal identifiers have been removed or disguised so the patient/person(s) described are not identifiable and cannot be identified through the details of the story.

## Statement of contribution

AG, CVW, and JW conceived the study. AG, EN, and CVW participated in the design. AG and EN participated in the acquisition of the data. AG, EN, and CVW participated in analysis of the data. All authors participated in interpretation of data, drafting and critical revision of the manuscript, and approved the final version.

## Figures and Tables

**Figure:**
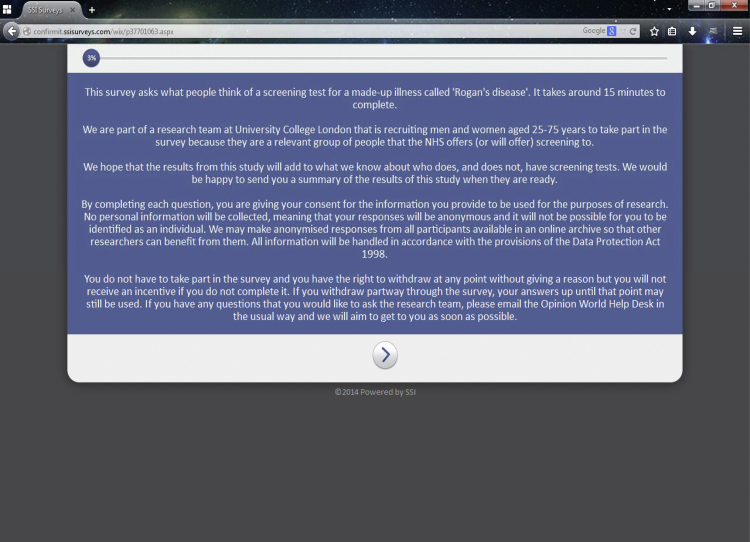


**Figure:**
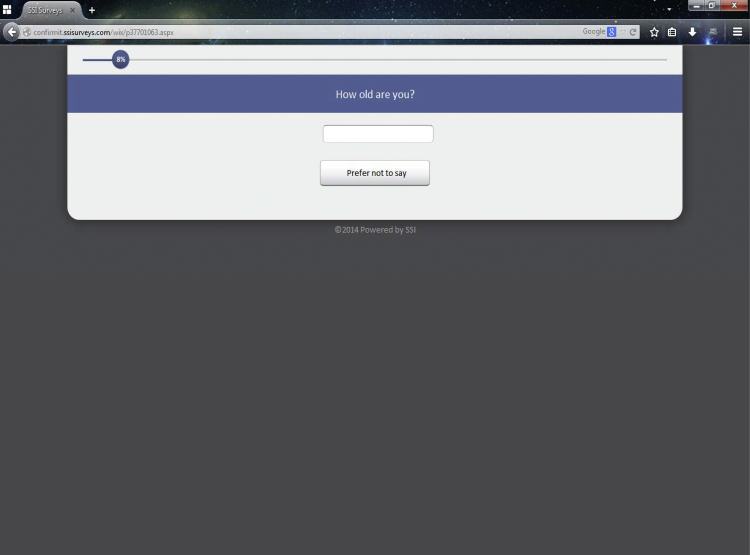


**Figure:**
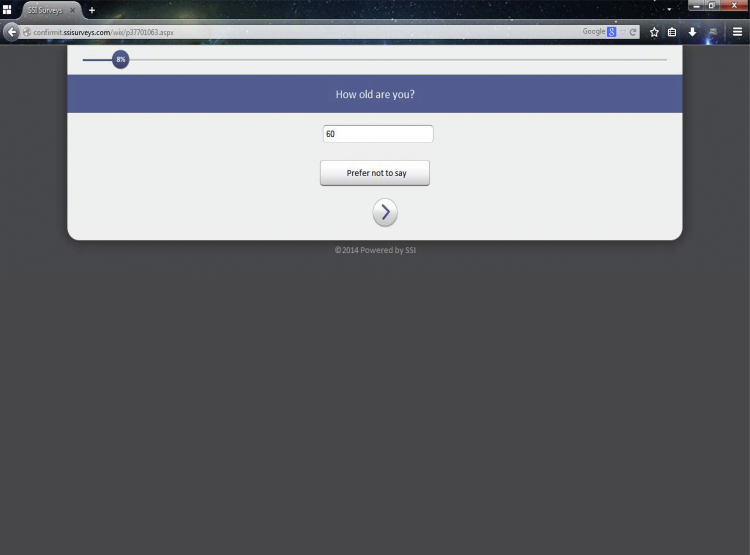


**Figure:**
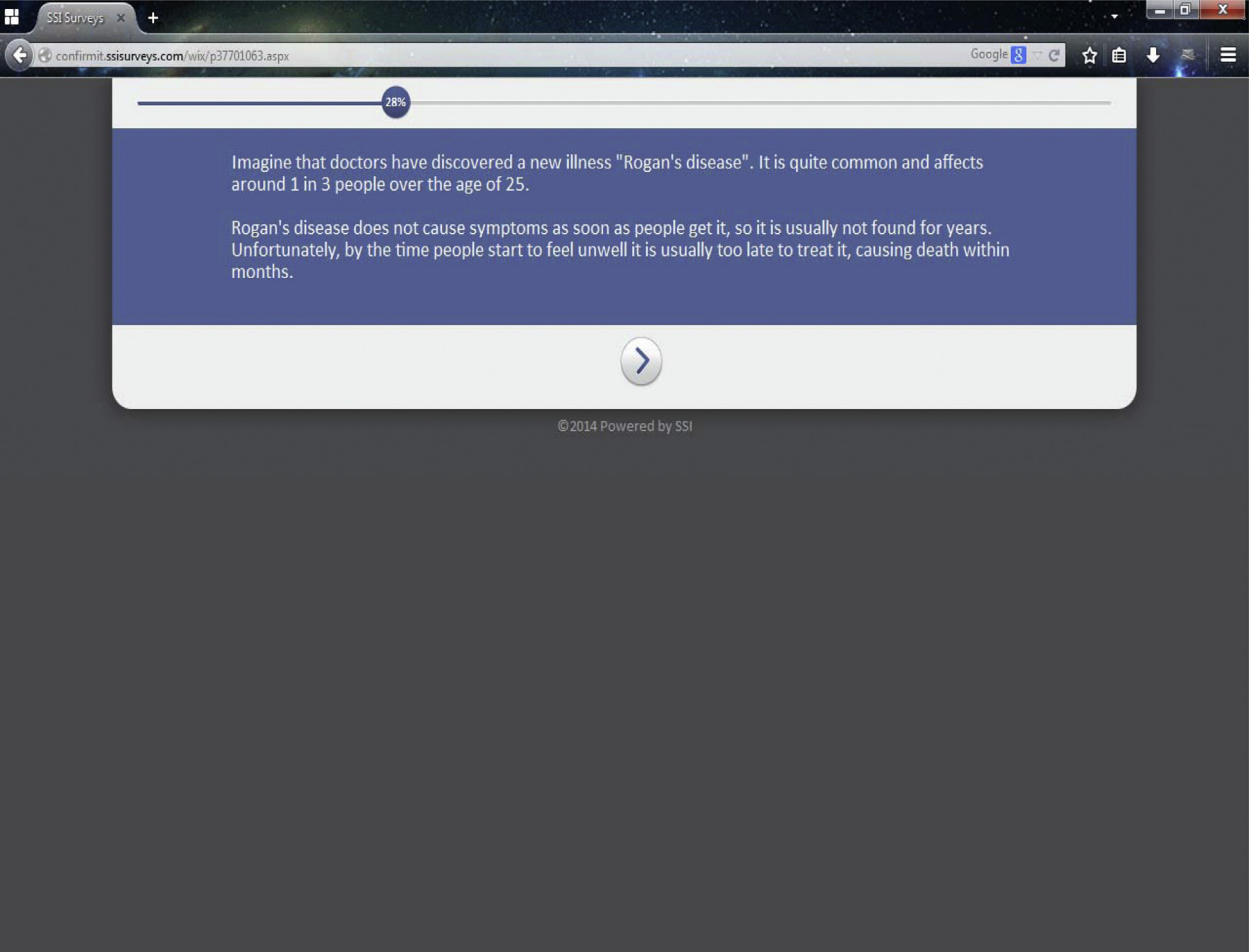


**Figure:**
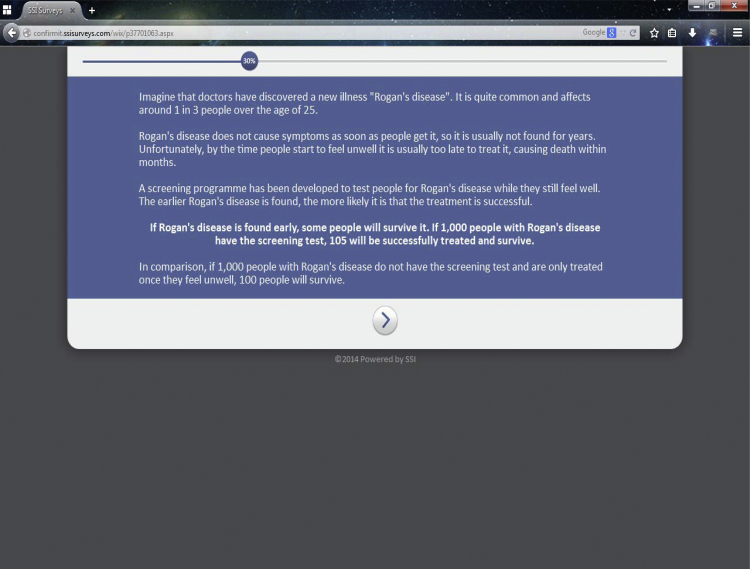


**Figure:**
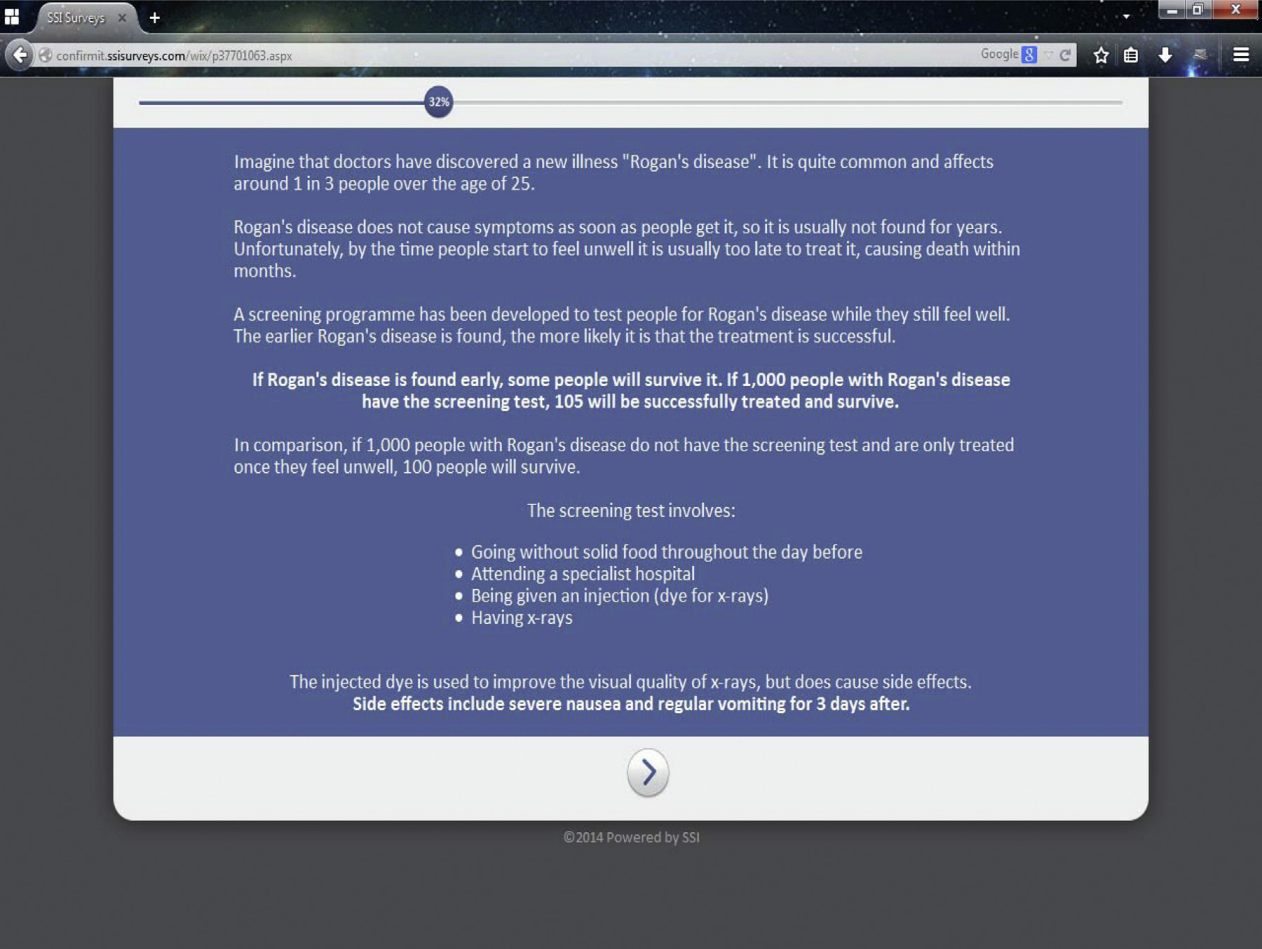


**Figure:**
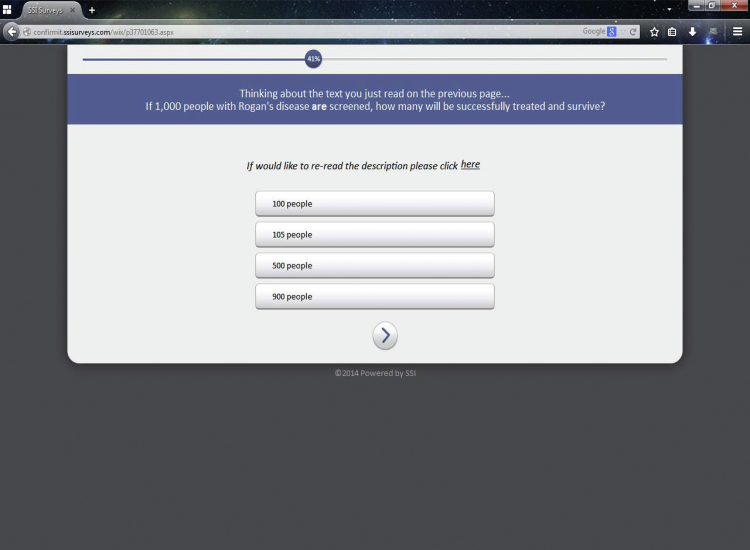


**Figure:**
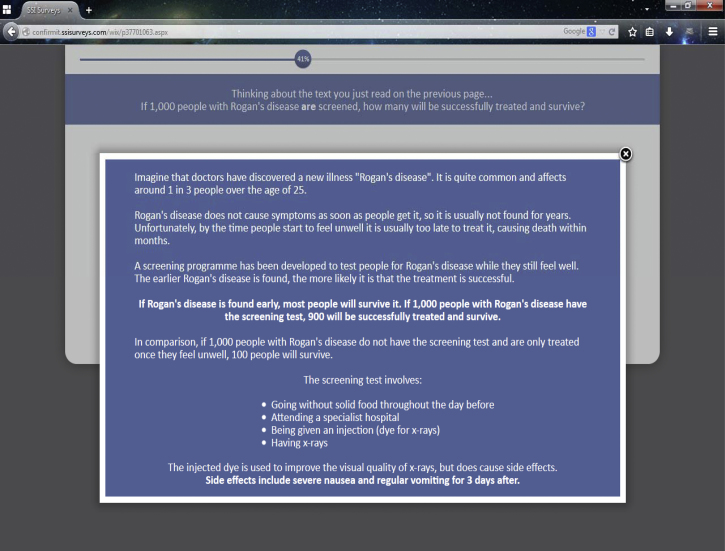


**Figure:**
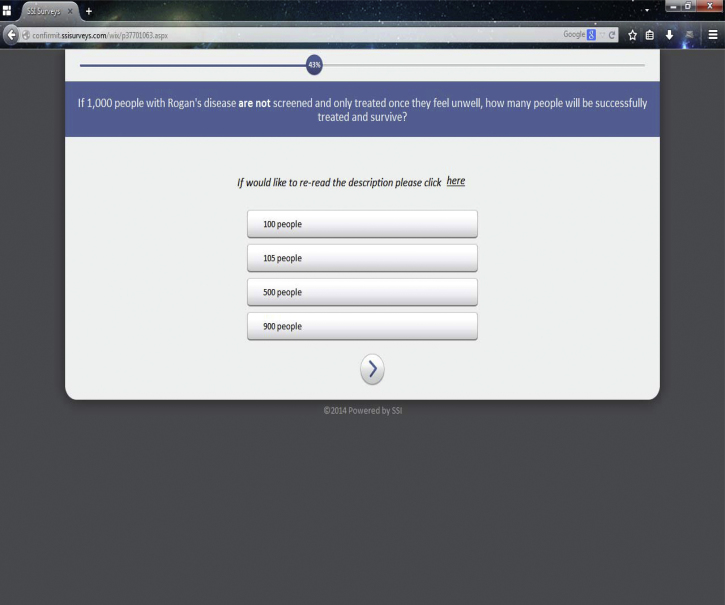


**Figure:**
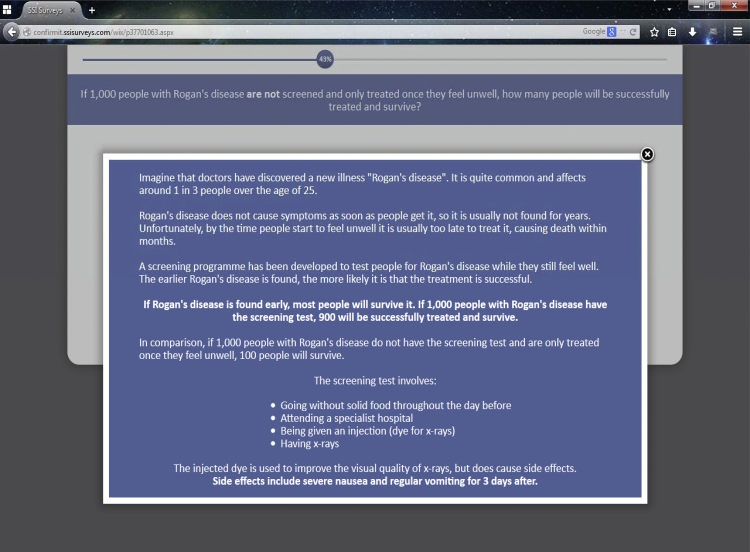


**Figure:**
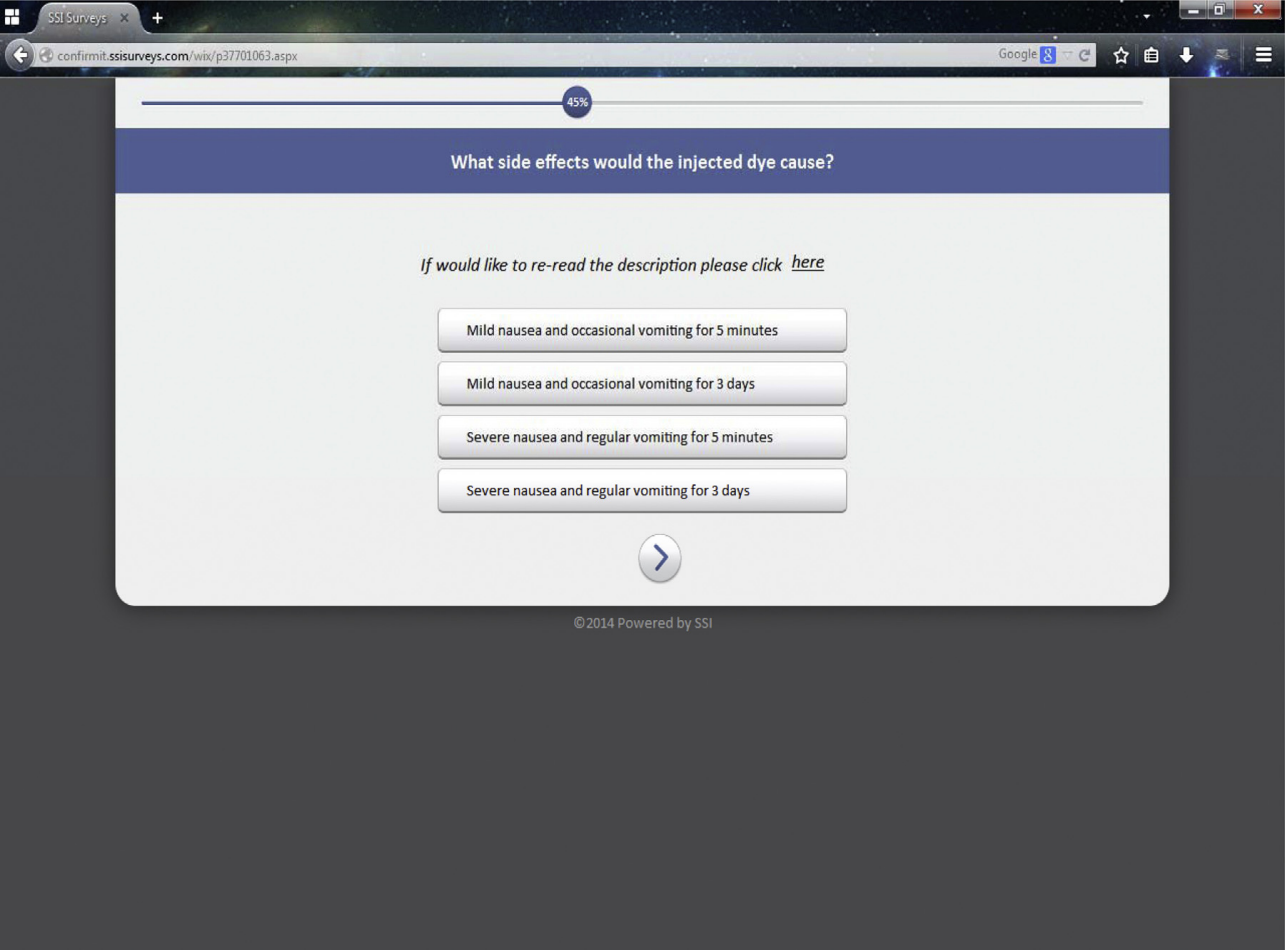


**Figure:**
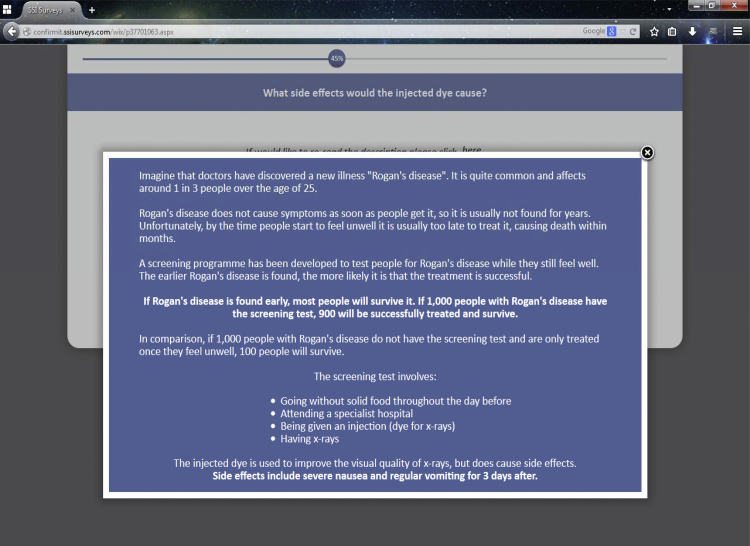


**Figure:**
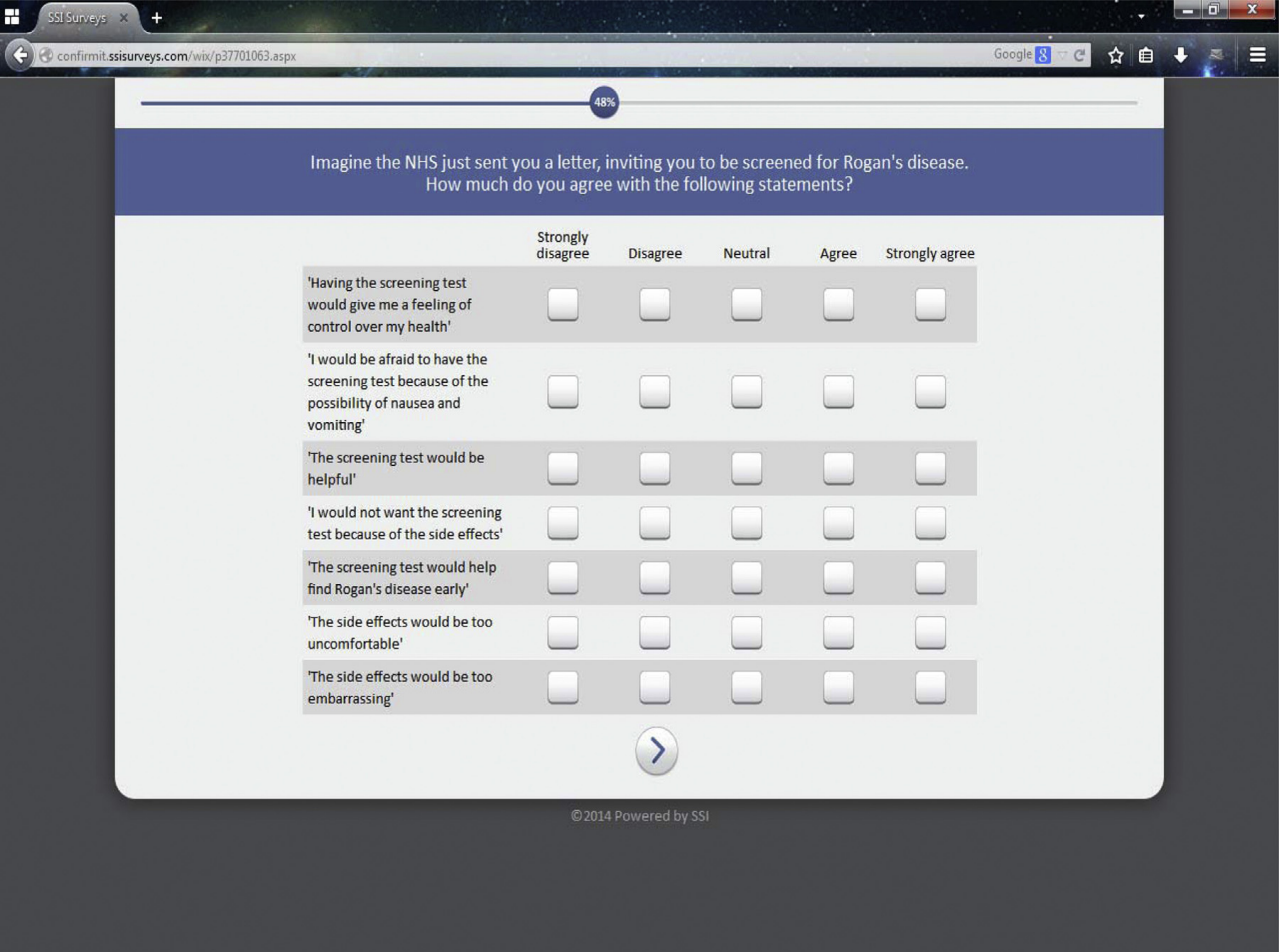


**Figure:**
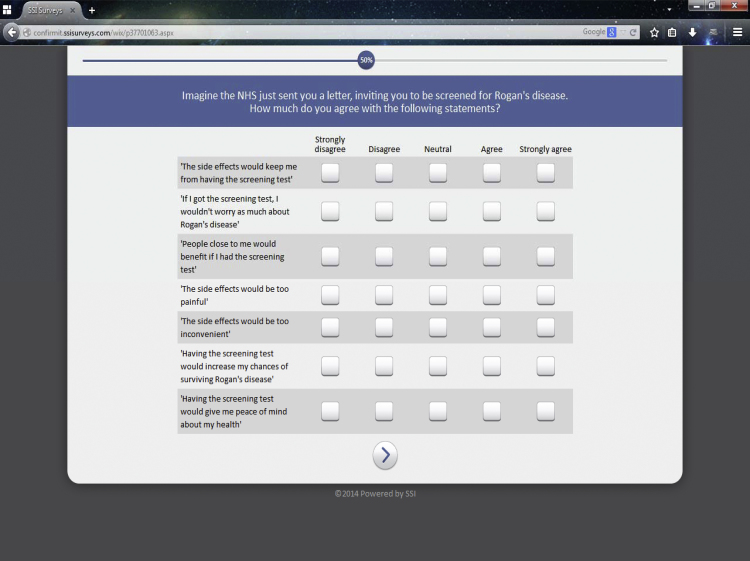


**Figure:**
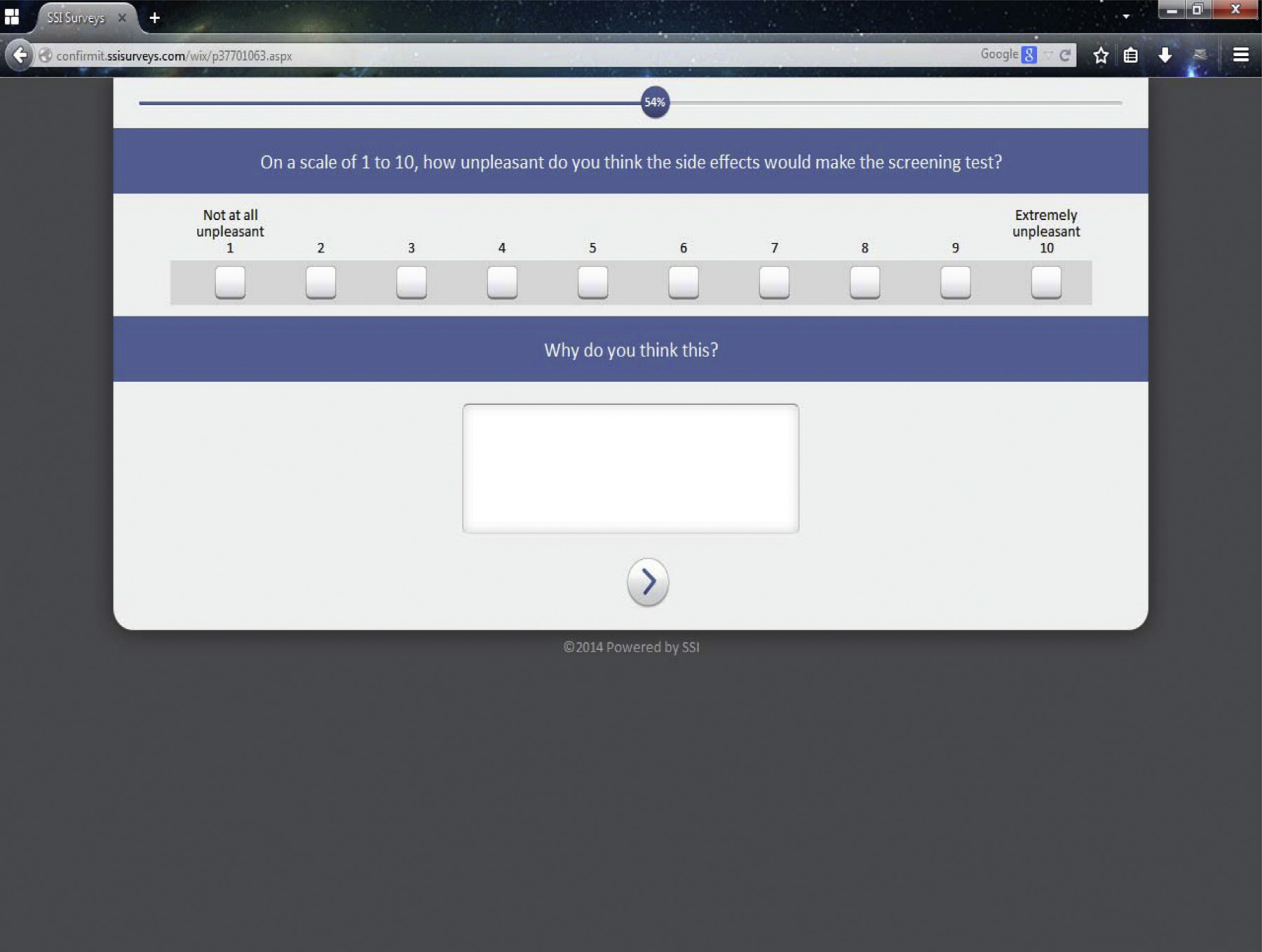


**Figure:**
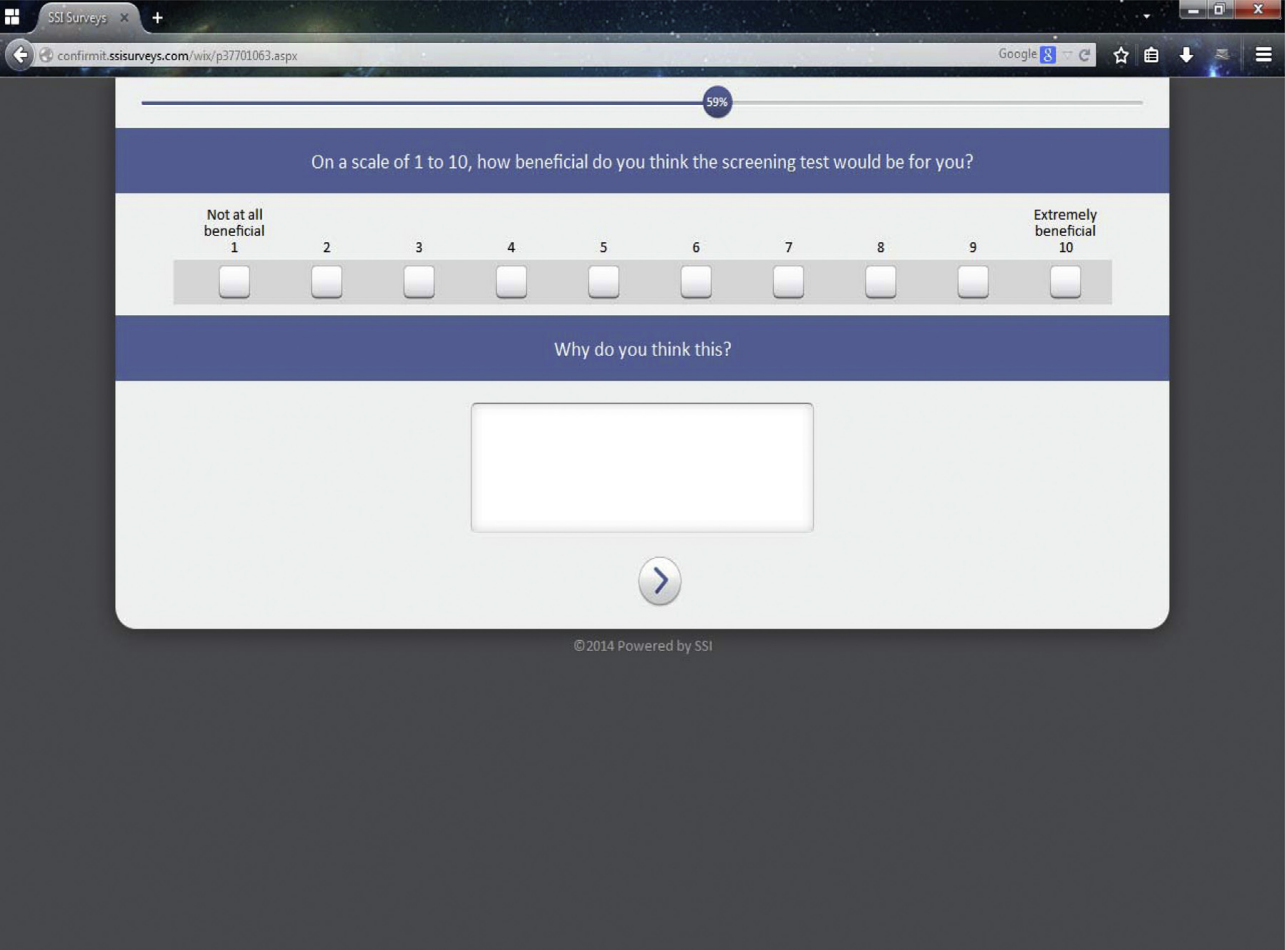


**Figure:**
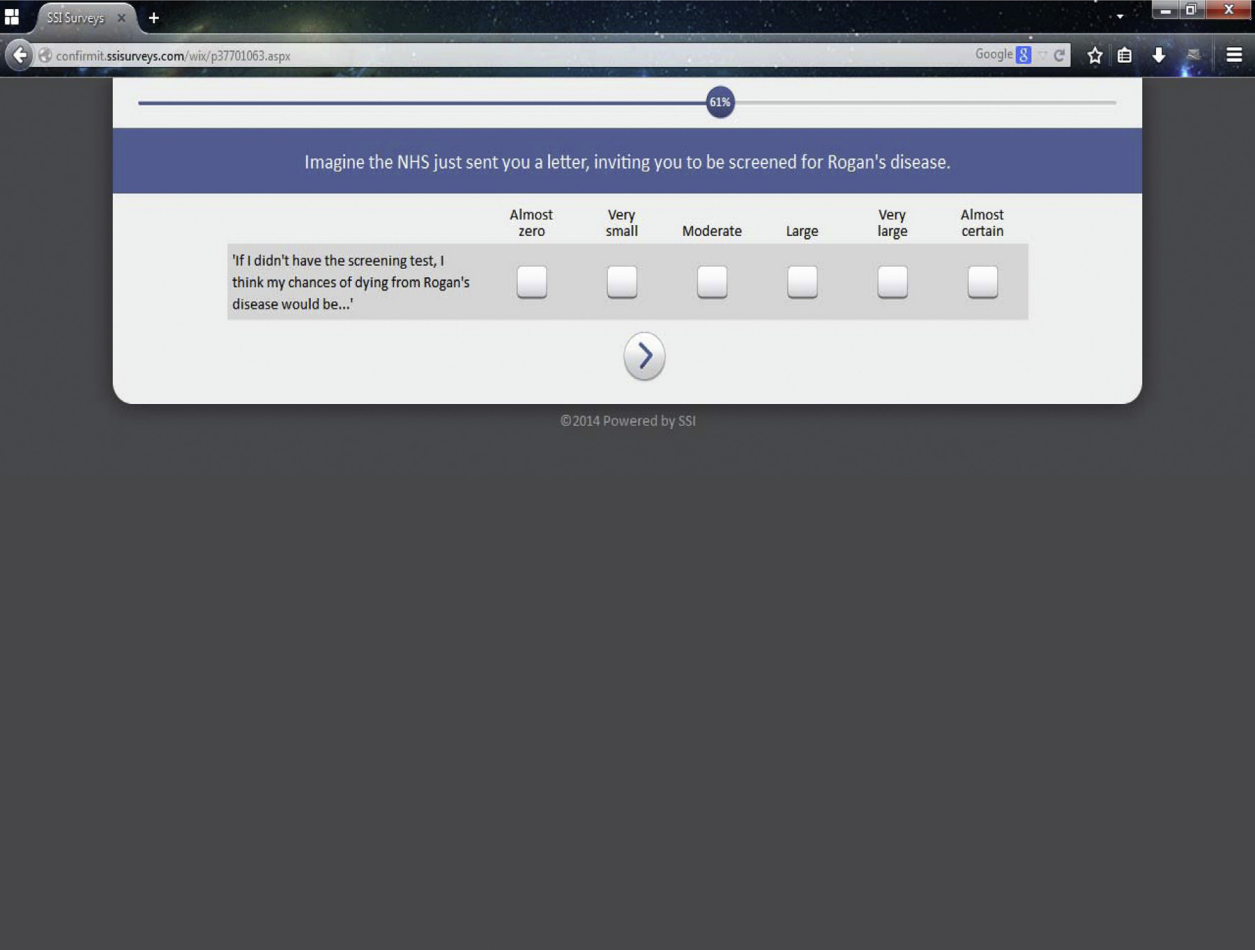


**Figure:**
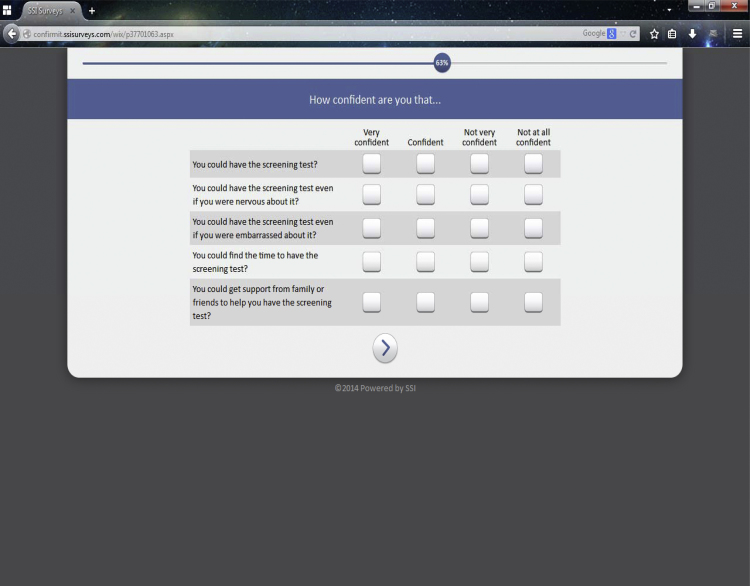


**Figure:**
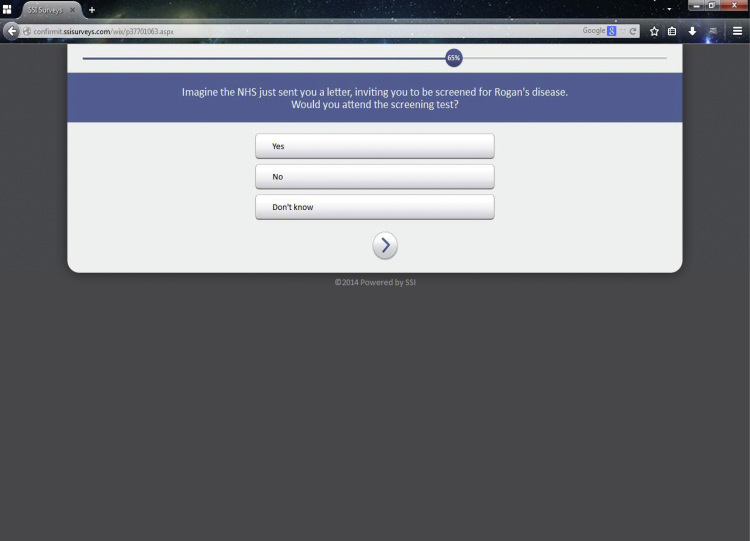


**Figure:**
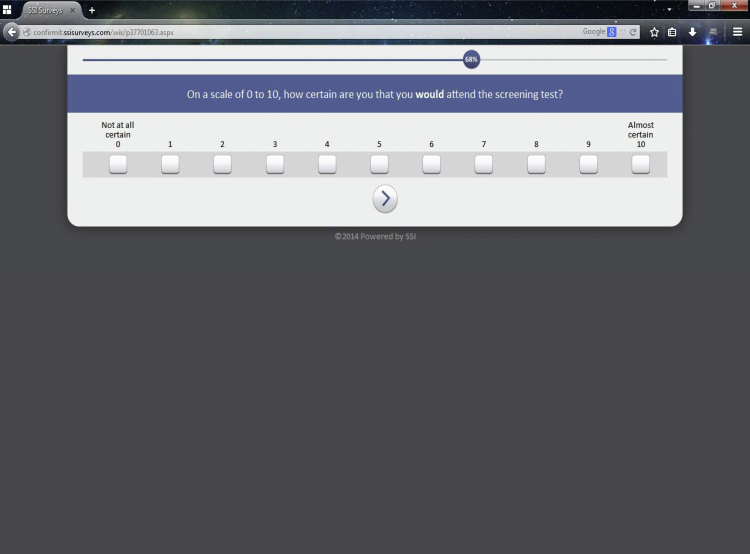


**Figure:**
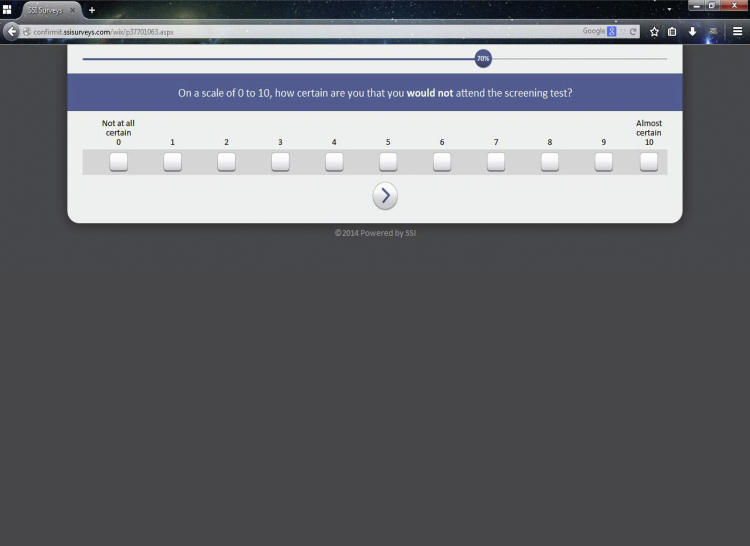


**Figure:**
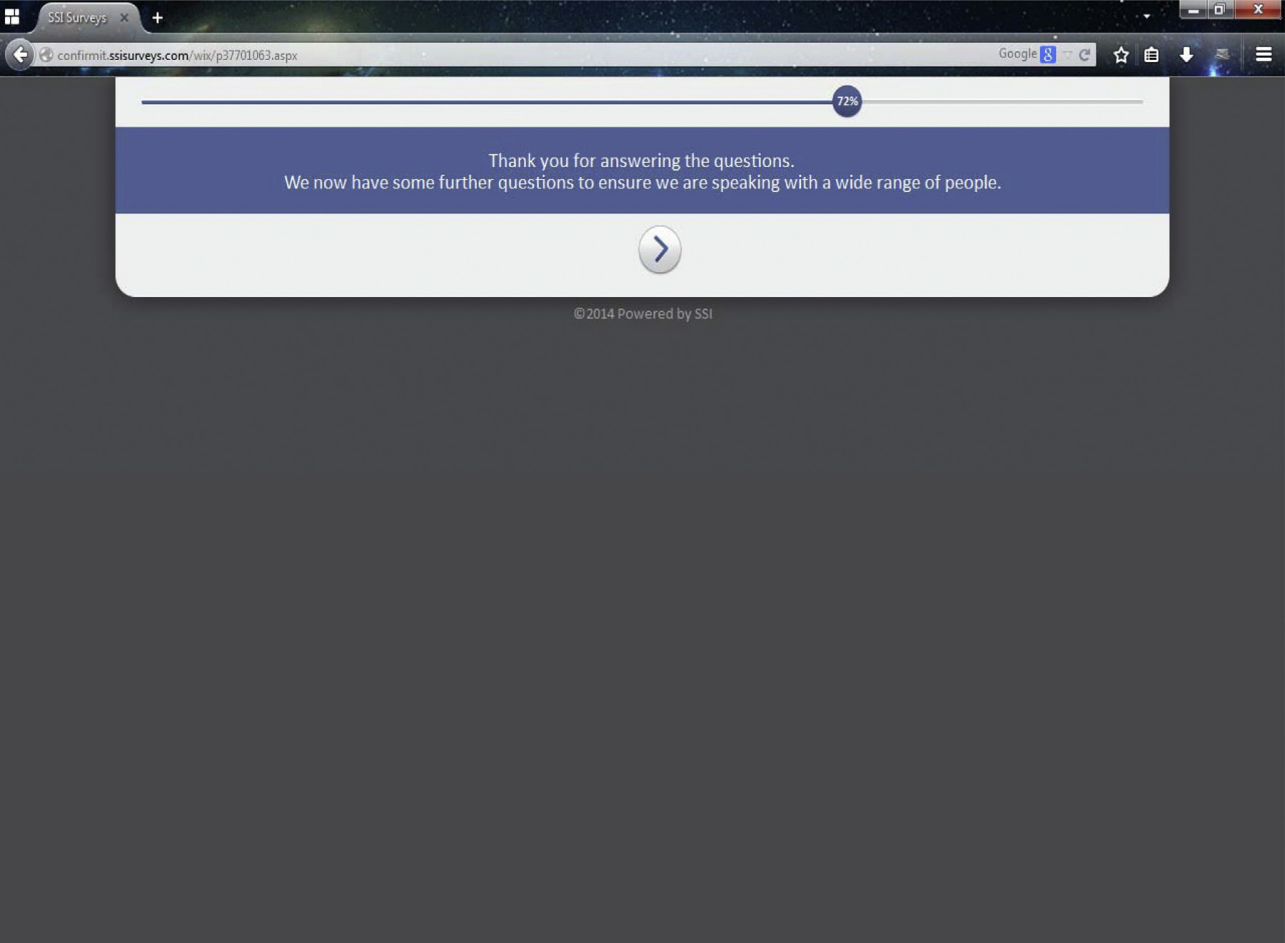


**Figure:**
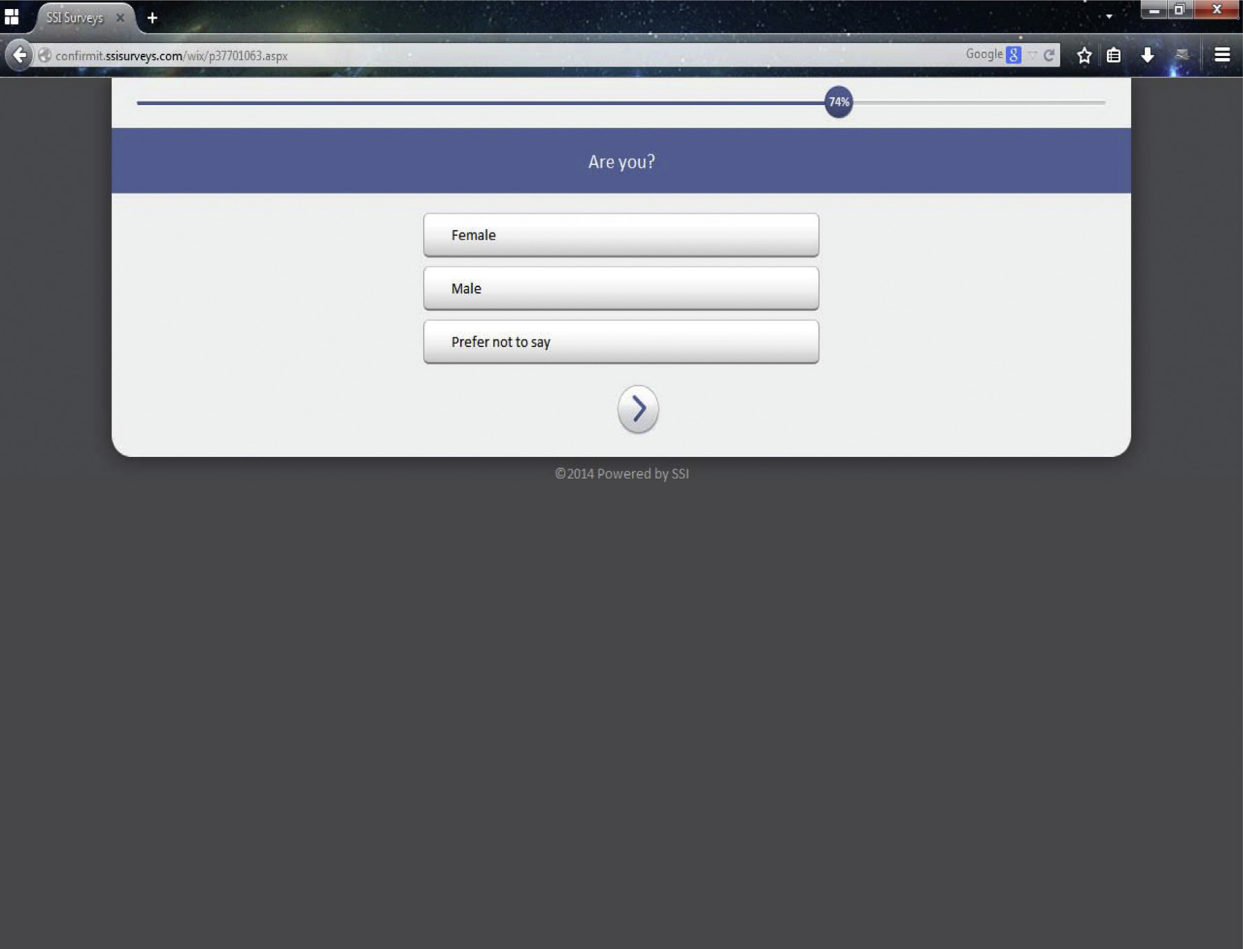


**Figure:**
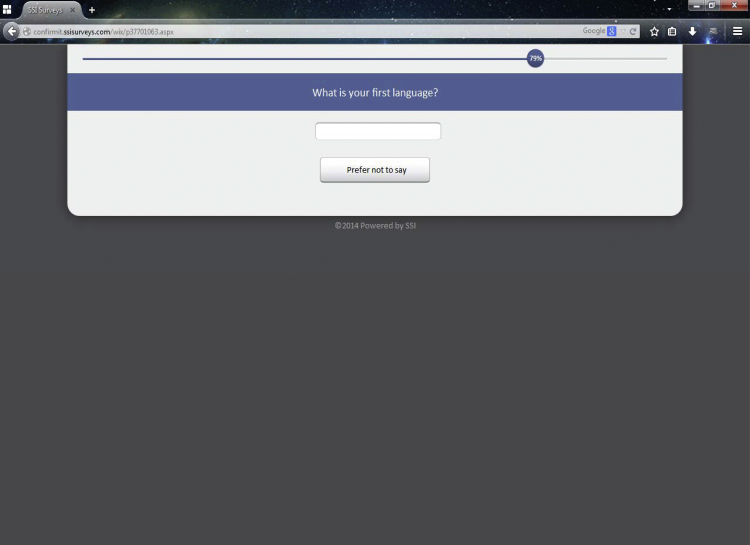


**Figure:**
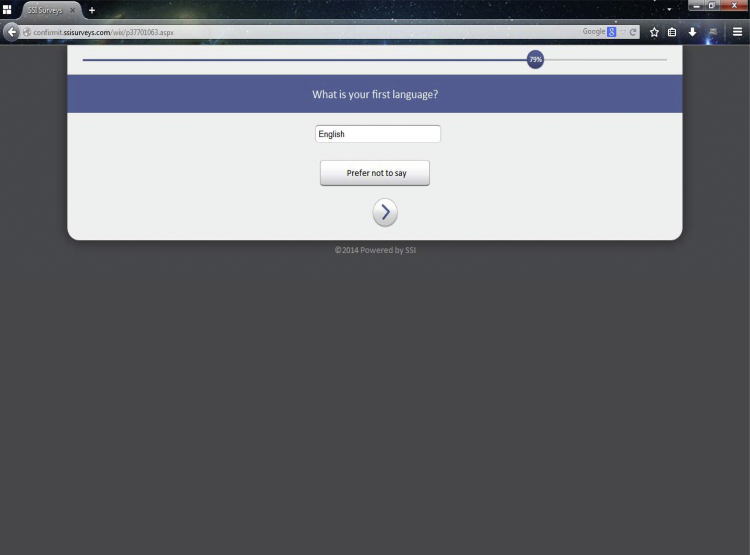


**Figure:**
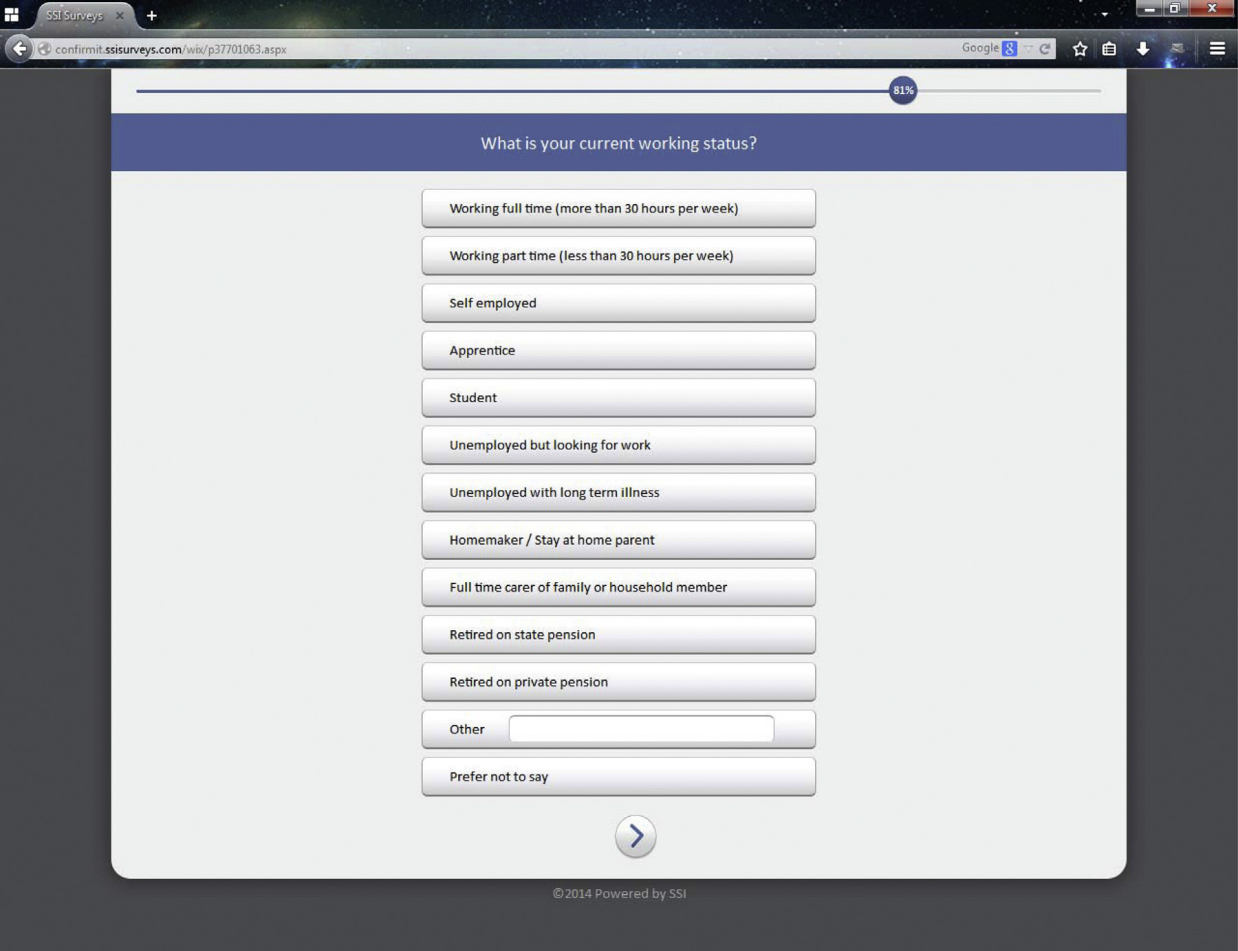


**Figure:**
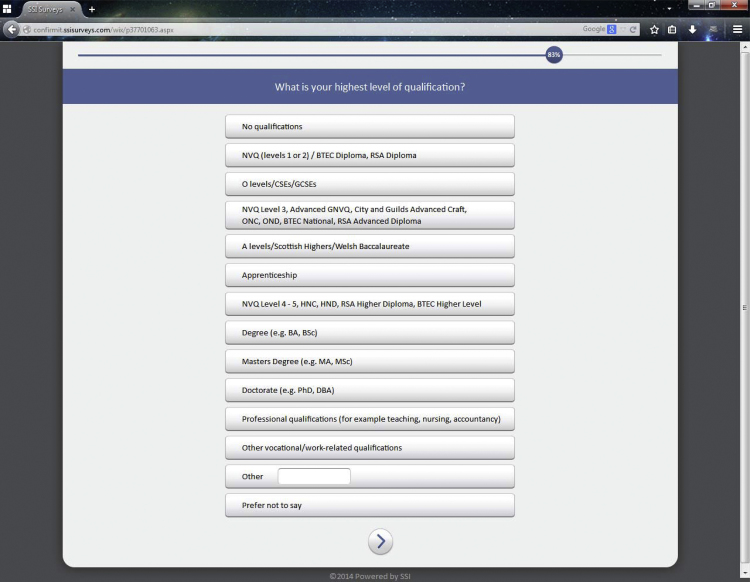


**Figure:**
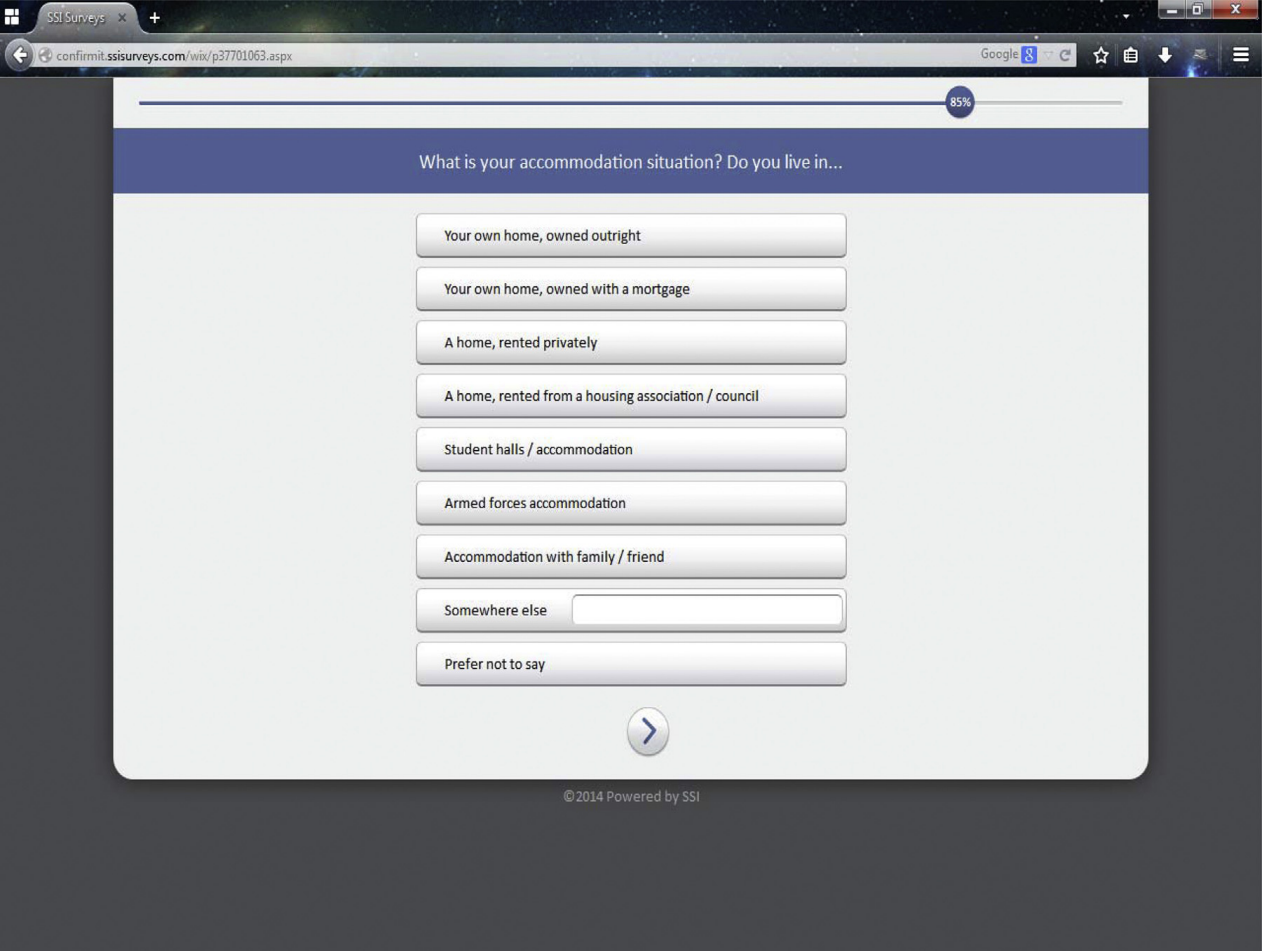


**Figure:**
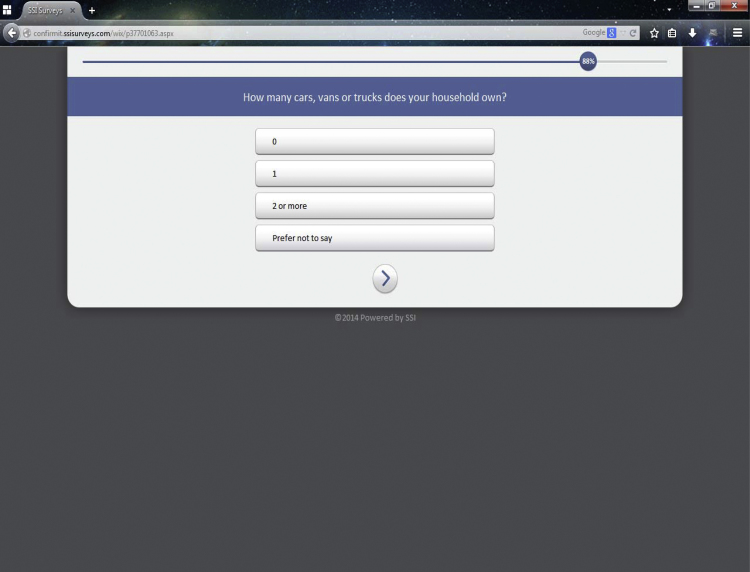


**Figure:**
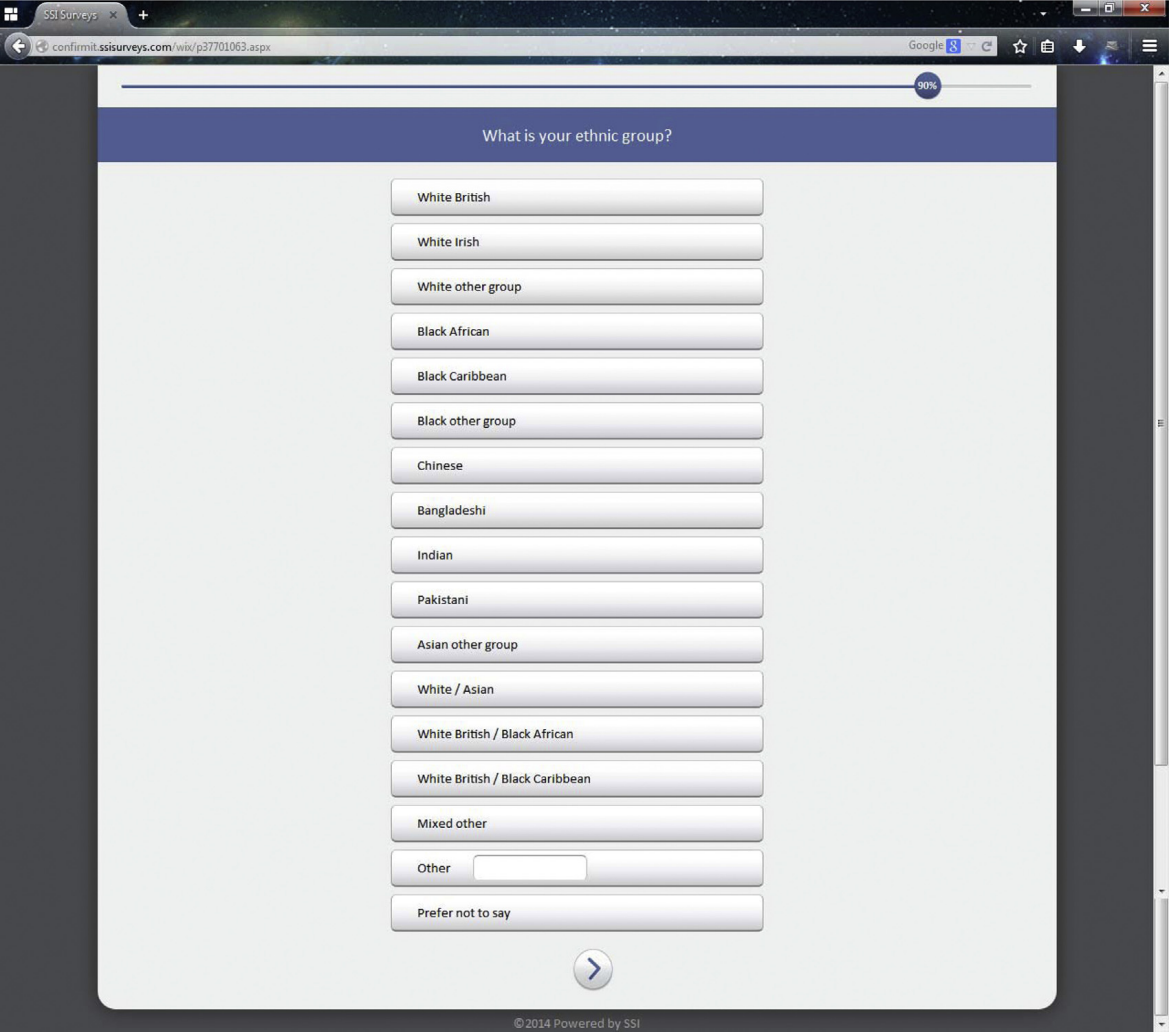


**Figure:**
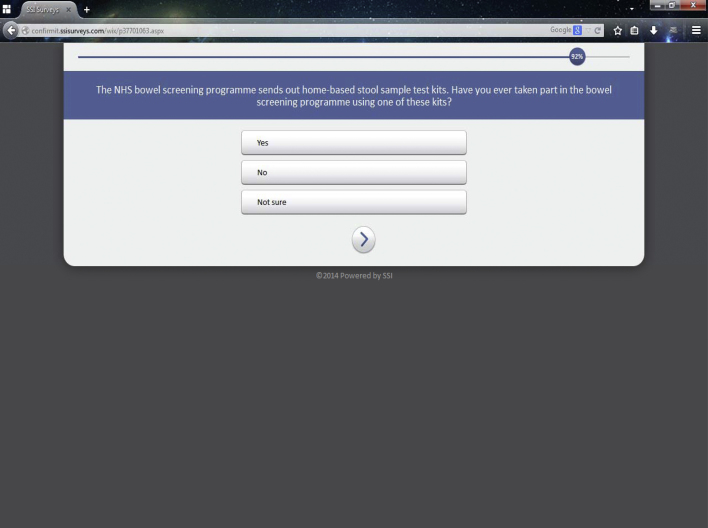


**Figure:**
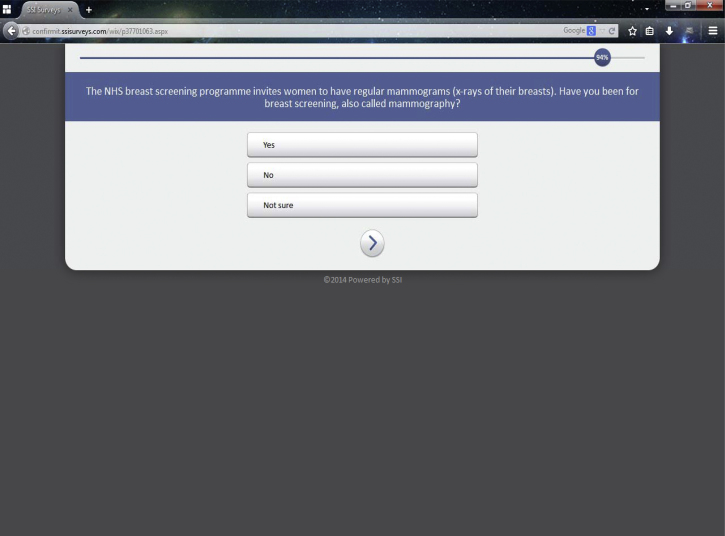


**Figure:**
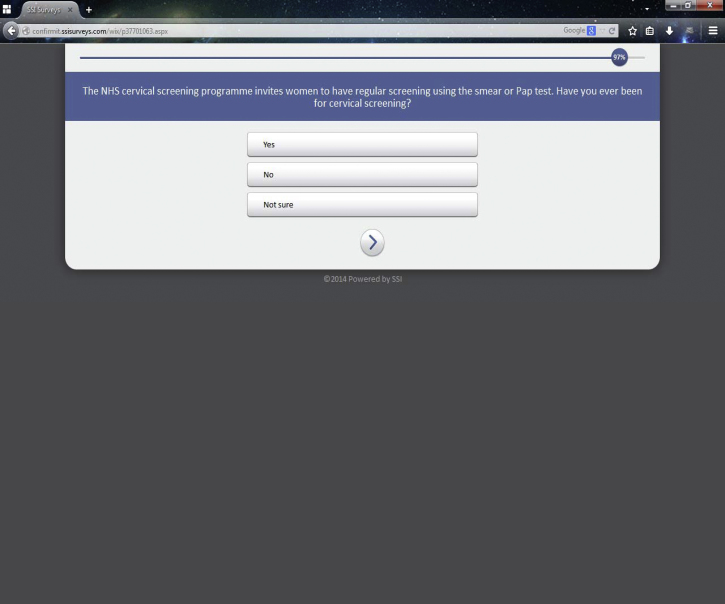


**Table 1:** Characteristics of analysed participants.

	High Benefit Low barrier	High benefit High barrier	Low benefit Low barrier	Low benefit High barrier	Total
	n = 52 *(%)*	n = 64 *(%)*	n = 56 *(%)*	n = 46 *(%)*	n = 218 (%)
Age					
Mean (standard deviation)	49.4 *(12.6)*	47.1 *(14.7)*	51.2 *(12.7)*	46.6 *(13.8)*	48.6 *(13.6)*

Gender					
Male	28 *(53.8)*	30 *(46.9)*	27 *(48.2)*	18 *(39.1)*	103 (47.2)
Female	24 *(46.2)*	34 *(53.1)*	29 *(51.8)*	28 *(60.9)*	115 (52.8)

Employment status					
Employed	31 *(59.6)*	38 *(59.4)*	36 *(64.3)*	31 *(67.4)*	136 *(62.4)*
Not employed/Retired	11 *(21.2)*	17 *(26.6)*	19 *(33.9)*	8 *(17.4)*	55 *(25.2)*
Other/Prefer not to say	10 *(19.2)*	9 *(14.1)*	1 *(1.8)*	7 *(15.2)*	27 *(12.4)*

Ethnicity					
White British	44 *(84.6)*	55 *(85.9)*	48 *(85.7)*	42 *(91.3)*	189 *(86.7)*
Other/Prefer not to say	8 *(15.4)*	9 *(14.1)*	8 *(14.3)*	4 *(8.7)*	29 *(13.3)*

First language					
English	51 (98.1)	61 (95.3)	53 (94.6)	45 (97.8)	210 (96.3)
Other/Prefer not to say	1 (1.9)	3 (4.7)	3 (5.4)	1 (2.2)	8 (3.7)

Socioeconomic status score					
0 (Least deprived)	33 (63.5)	32 (50.0)	34 (60.7)	27 (58.7)	126 (57.8)
1	12 (23.1)	24 (37.5)	15 (26.8)	12 (26.1)	63 (28.9)
2	6 (11.5)	7 (10.9)	7 (12.5)	6 (13.0)	26 (11.9)
3 (Most deprived)	1 (1.9)	1 (1.6)	0 (0.0)	1 (2.2)	3 (1.4)

CRC screening experience^a^					
Yes	(75.0)	11 (73.3)	12 (75.0)	7 (70.0)	39 (73.6)
No	3 (25.0)	4 (26.7)	4 (25.0)	3 (30.0)	14 (26.4)
Not sure	0 (0.0)	0 (0.0)	0 (0.0)	0 (0.0)	0 (0.0)
Not applicable	40 (N/A)	49 (N/A)	40 (N/A)	36 (N/A)	165 (N/A)

Breast cancer screening experience					
Yes	12 (85.7)	13 (100.0)	13 (72.2)	13 (100.0)	51 (87.9)
No	2 (14.3)	0 (0.0)	5 (27.8)	0 (0.0)	7 (12.1)
Not sure	0 (0.0)	0 (0.0)	0 (0.0)	0 (0.0)	0 (0.0)
Not applicable	38 (N/A)	51 (N/A)	38 (N/A)	33 (N/A)	160 (N/A)

Cervical cancer screening experience					
Yes	23 *(95.8)*	28 *(82.4)*	28 *(96.6)*	20 *(71.4)*	99 *(86.1)*
No	1 *(4.2)*	6 *(17.6)*	1 *(3.4)*	7 *(25.0)*	15 *(13.0)*
Not sure	0 *(0.0)*	0 *(0.0)*	0 *(0.0)*	1 *(3.6)*	1 *(0.9)*
Not applicable	28 (N/A)	30 (N/A)	27 (N/A)	18 (N/A)	103 (N/A)

Perceived chance of dying of Rogan’s disease (in the absence of screening)					
Almost zero	0 (0.0)	0 (0.0)	2 (3.6)	3 (6.5)	5 (2.3)
Very small	7 (13.5)	10 (15.6)	18 (32.1)	17 (37.0)	52 (23.9)
Moderate	16 (30.8)	25 (39.1)	16 (28.6)	14 (30.4)	71 (32.6)
Large	16 (30.8)	11 (17.2)	10 (17.9)	8 (17.4)	45 (20.6)
Very large	12 (23.1)	12 (18.8)	7 (12.5)	3 (6.5)	34 (15.6)
Almost certain	1 (1.9)	6 (9.4)	3 (5.4)	1 (2.2)	11 (5.0)

Self-efficacy					
Mean (standard deviation)	7.8 (3.2)	8.6 (2.9)	8.7 (3.0)	11.0 (3.6)	8.9 (3.3)

a Screening experience percentages are for age- and gender-applicable subgroups.

**Table 2:** Crude means and standard deviations for perceived benefit and barrier scores for all four conditions.

	Possible combinations of benefits and barriers levels			
	High benefits Low barriers	High benefits High barriers	Low benefits Low barriers	Low benefits High barriers
	(n = 52)	(n = 64)	(n = 56)	(n = 46)
Perceived benefits	30.4 (4.3)	29.7 (3.8)	26.7 (4.7)	24.2 (5.4)
Perceived barriers	12.6 (6.0)	18.2 (7.3)	13.5 (5.4)	21.5 (7.7)

## Footnotes

1 Three single items were devised to assess i) perceived barriers, ii) perceived benefits, and iii) “intention certainty” as part of piloting the survey. These were also included in the main study but after preliminary analysis of the data, it was unclear how to interpret the distributions of data. Hence, these ad-hoc items were considered to lack sufficient face validity and were not used for further analysis (particularly since superior measures of perceived barriers and benefits were available).
